# Unilateral primitive hydatid cyst with surgical resection of the scrotum: a case report

**DOI:** 10.1186/1752-1947-7-109

**Published:** 2013-04-19

**Authors:** Ahmed Amine Bouchikhi, Youssef Alaoui Lamrani, Mohamed Fadl Tazi, Soufiane Mellas, Jalaledine Elammmari, Abdelhak Khallouk, Mohammed Jamal Elfassi, Moulay Hassan Farih

**Affiliations:** 1 Urology Department, University Hospital of Fez, Fez, Morocco; 2 Rue Zag, Rce Andalous III, Quarier Al-Wafe, Fès 30070, Morocco

## Abstract

**Introduction:**

Hydatid disease remains a public health problem in many Mediterranean countries. Liver and lung localizations are the most common. Renal hydatid cysts represent 2 percent to 4 percent of the visceral forms of this disease. To the best of our knowledge a scrotal location has only previously been described in five papers in the literature, all being secondary localizations. In this paper, we report a case of a primitive scrotal hydatid cyst.

**Case presentation:**

A Moroccan man aged 29 years old presented to our facility with scrotal pain. A clinical examination identified a painless scrotal mass. The results of a scrotal ultrasound showed intra-scrotal cystic formations with different sizes associated with scrotal effusion of average abundance. Chest cavity and abdominal computed tomography scans did not reveal any other localizations. Our patient benefited from surgical protruding dome resection. A partial cysto-pericystectomy was realized. The first stage consisted of injecting a scolicide solution; hydrogen peroxide is the most commonly used agent. This is injected into the cystic cavity and retained for 10 minutes. This process allows for sterilization of the cyst while avoiding the risk of rupture and transmission of the hydatid liquid into the circulation. After 10 minutes, the cystic contents are removed by suction. The cyst is then opened, and the endocyst containing the hydatid membrane and daughter vesicles are removed. It is of note that our patient did not receive any additional medical treatment. Our diagnosis was made using an imaging approach and was confirmed during surgery.

**Conclusions:**

Ultrasound is often the key diagnostic approach for cases of a scrotal hydatid cyst. Treatment is primarily surgical, aiming for resection of the protruding dome via a longitudinal scrotectomy.

## Introduction

Hydatid disease is a cosmopolitan anthropozoonosis common in human and many mammals due to development in the body of *Echinococcus granulosus* (EG), the larval form of the tapeworm known as the dog tapeworm. It is endemic in many farming countries. To the best of our knowledge, a scrotal localization for a hydatid cyst has only been described five times previously in the literature, with these cases all being secondary localizations [1]. We present a case of a primitive hydatid cyst localized in the scrotum of a 29-year-old patient. The diagnosis was made by ultrasound, and was confirmed by surgery. We discuss the pathophysiology, epidemiology, clinical and therapeutic aspects of this disease through the study of this case and a review of the literature.

## Case presentation

**Figure 1:**
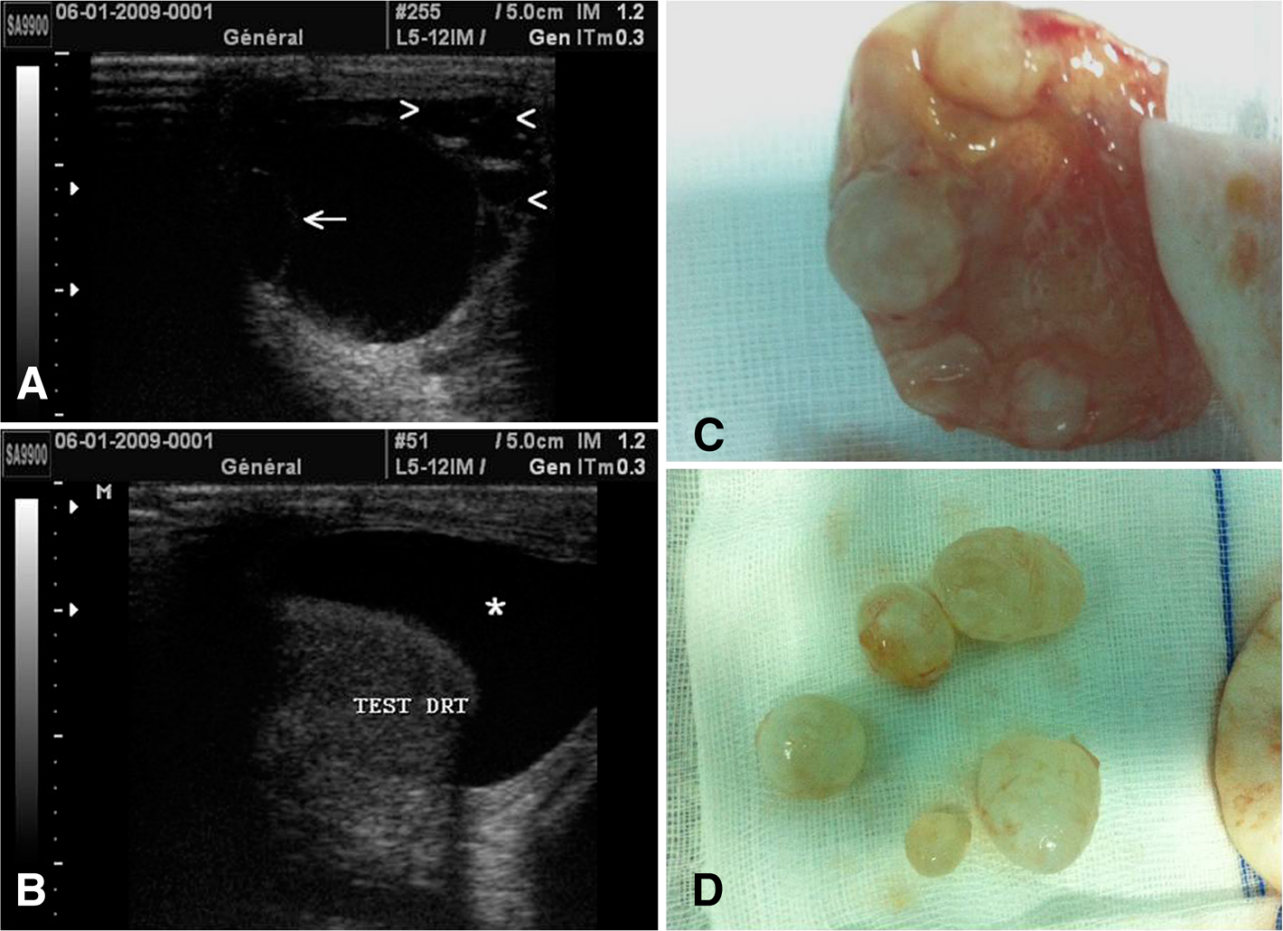
**Ultrasound images showing right cystic lesions (A, B).** The largest cyst contained a daughter vesicle (arrow); this was associated with hydrocele of average abundance (star). Hydatid larvae with daughter vesicles are shown in **(C)** and **(D)**.

A 29-year-old Moroccan man with no relevant medical history presented to our urology department due to an increased scrotal volume lasting several months. Our patient was in a good general condition; the results of a clinical examination detected a painless scrotal mass in a febrile patient with a temperature of 39°C. An ultrasound scan showed right-sided cystic lesions of different sizes; while the largest cyst contained a daughter vesicle, this was associated with hydrocele of average abundance (Figure 1A,B).

A study of the chest and abdominal cavities using computed tomography (CT) scans did not reveal any other locations.

Our patient benefited from surgical protruding dome resection via a longitudinal scrotectomy. A partial cysto-pericystectomy was performed. The first stage consists of injecting a scolicide solution; hydrogen peroxide is the most commonly used agent; it is injected in the cystic cavity and retained for 10 minutes. This process allows for sterilization of the cyst while avoiding the risk of rupture and transmission of the hydatid liquid into the circulation. After 10 minutes, the cystic contents are removed by suction. The cyst is then opened, and the endocyst containing the hydatid membrane and daughter vesicles are removed. It is of note that our patient did not receive any additional medical treatment (Figure 1C,D). He showed good clinical and imaging results at six-month follow-up.

## Discussion

Hydatid disease is endemic in North Africa, some countries of the Mediterranean basin, New Zealand, Australia, Asia, and America. It constitutes a genuine public health problem [2,3]. The geographical distribution of EG is correlated with economic and cultural levels. Thus, the prevalence of hydatidosis is variable. Kenya is the country where the prevalence is highest (200 cases per 100,000 population per year). The Maghreb is an intermediate zone; here, the prevalence of hydatidosis is 15 cases per 100,000 population per year in Tunisia, and 8 cases per 100,000 population per year in Morocco [4,5].

EG is hosted in the small intestine of carnivores (dogs, wolves and other canines) that represent the definitive hosts. Eggs are voided with feces. They are ingested by herbivores, often sheep, which are the intermediate hosts.

Once the hexacanth embryo is released and penetrates the intestinal wall it often enters the portal venous circulation to reach the liver. A total of 60 percent to 75 percent of cases exhibit liver forms of the disease, whereas 15 percent to 30 percent migrate to the lungs through the hepatic veins. In exceptional cases the larva might migrate to another organ through the blood or lymphatic system [4,6-8].

Ingestion of a hydatid cyst by a canine leads to the release of larvae into the intestine. At this stage, the larvae become adult worms. Human infection occurs by accidental ingestion of *E. granulosus* eggs, by absorption of contaminated food or contact with dog feces [4,7,9]. This explains the high prevalence of hydatid cysts in rural areas [7,10].

The liver and lung are the most commonly infected organs with respective rates of 65 percent and 25 percent [11]. The most affected urogenital tract is the kidney with 2 percent to 4 percent of all localizations. However, to the best of our knowledge a scrotal localization has only been reported in the literature five times previously, with these cases all being secondary localizations [1].

Contamination of the scrotum would be secondary to rupture of an intra-abdominal cyst or primitive cyst, as was the case in our patient; this was achieved through the blood or lymphatic system [12].

Eosinophilia is interesting only from a point of view of guiding the diagnosis, while the hydatid serology has an important false negative ratio [13].

Hydatidosis imaging is specific, especially on ultrasound; the cyst may be single or multi-loculated, with homogeneous or heterogeneous fluid content presenting xa limited thin or thick membrane. Imaging might demonstrate a vesicle membrane disjointing or daughter vesicle that is also visible on CT and magnetic resonance imaging (MRI). Imaging studies allow objectifying of fluid-fluid levels, with intra-cystic parietal enhancement reflecting altered or infected cysts [14,15]. Calcification and partition of the wall can be identified on ultrasound and CT. Imaging should to be used to find out other localizations including hydatid liver, lung, kidney or bone.

The scrotal ultrasound of our patient clearly demonstrated right intra-scrotal cystic formations of different sizes; the largest one hosted a daughter vesicle. This was associated with scrotal effusion of average abundance (Figure 1B).

The prognosis depends on early diagnosis and surgery. Long-term close follow-up of patients is important to prevent recurrence. The treatment for hydatid cysts is based on the surgical evacuation of their contents and sterilization of the residual cavities.

Our patient benefited from surgical resection of the protruding dome; this included longitudinal scrotectomy sterilization of the cavity. There was a good clinical and radiological result at six-month follow-up.

The majority of authors in the literature reported the choice of treatment was protruding dome resection. This treatment has been shown to be efficient, with good post-operative results.

Medical treatment is based on mebendazole; albendazole is still controversial, and does not seem to reduce the risk of recurrence [3,10,16,17].

## Conclusions

Hydatidosis of the scrotum has to be considered in cases of scrotal swelling in endemic countries. Ultrasound is often the key diagnostic approach for hydatid cyst of the scrotum. Treatment is primarily surgical, aiming for resection of the protruding dome via longitudinal scrotectomy.

## Consent

Written informed consent was obtained from the patient for publication of this case report and any accompanying images. A copy of the written consent is available for review by the Editor-in-Chief of this journal.

## Competing interests

The authors state that they have no competing interests.

## Authors’ contributions

AAB was the principal author and major contributor in writing the manuscript. MFT and YAL collaborated in writing the manuscript, analyzed and interpreted the data from our patient and reviewed the literature. MFT, JE, AK, SM, MJE and MHF read and corrected the manuscript. All authors read and approved the final manuscript.
